# Supplementary material for: Can telehealth expansion boost health care utilization specifically for patients with substance use disorders relative to patients with other types of chronic disease?
Source: PLoS One. 2024 Apr 1;19(4):e0299397. doi: 10.1371/journal.pone.0299397 (PMC10984462; doi:10.1371/journal.pone.0299397)
Supplement: S1 Appendix — (DOCX) [file pone.0299397.s001.docx]

**S1 Appendix. Sample Construction**

| **Inclusion/Exclusion Criteria for Study Population** | **Unique Individuals** |
| --- | --- |
| 1. Population: Individuals ages 19-64 enrolled in June 2019 via parent/caretaker or childless adult eligibility | 284,418 |
| 1. Without any eligibility June 2019-December 2020 due to pregnancy | 273,102 |
| 1. Continuous enrollment from June 2019 – December 2020 | 143,992 |
| 1. Diagnosis of SUD in any position on outpatient, inpatient, or emergency department claim, December 2018-May 2019; OR Diagnosis of type 2 diabetes in any position on outpatient, inpatient, or emergency department claim, December 2018-May 2019 | SUD 17,336  Diabetes 8,499 |

| **Inclusion/Exclusion Criteria for Comparison Population** | **Unique Individuals** |
| --- | --- |
| 1. Population: Individuals ages 19-64 enrolled in June 2018 via parent/caretaker or childless adult eligibility | 290,695 |
| 1. Without any eligibility June 2018-December 2019 due to pregnancy | 277,590 |
| 1. Continuous enrollment from June 2018 – December 2019 | 112,286 |
| 1. Diagnosis of SUD in any position on outpatient, inpatient, or emergency department claim, December 2017-May 2018; OR Diagnosis of type 2 diabetes in any position on outpatient, inpatient, or emergency department claim, December 2017-May 2018 | SUD 13,546  Diabetes 7,235 |

Abbreviations: SUD, substance use disorder
