# Supplementary material for: Can telehealth expansion boost health care utilization specifically for patients with substance use disorders relative to patients with other types of chronic disease?
Source: PLoS One. 2024 Apr 1;19(4):e0299397. doi: 10.1371/journal.pone.0299397 (PMC10984462; doi:10.1371/journal.pone.0299397)
Supplement: S2 Appendix — (DOCX) [file pone.0299397.s002.docx]

**S2 Appendix. Comparison of analytic sample to population**

**Table 1. Comparison of analytic sample to population of Wisconsin Medicaid beneficiaries enrolled June 2019 and with continuous enrollment 06/01/2019–12/31/2020 via Parent/Caretaker or Childless Adult eligibility**

|  | **Not Continuously Enrolled** | | **Continuously Enrolled** | | **SUD Cohort** | | | **Diabetes Cohort** | | |
| --- | --- | --- | --- | --- | --- | --- | --- | --- | --- | --- |
| N=unique subjects | 129,113 | 100.00% | 143,992 | 100.00% | 17,336 | 100.00% | 8,499 | | 100.00% | |
| **Eligibility Category** |  |  |  |  |  |  |  | | |  |
| Childless Adults | 66,741 | 51.69% | 82,434 | 57.25% | 11,841 | 68.31% | 5,373 | | | 63.22% |
| Parents/Caretakers | 62,372 | 48.31% | 61,558 | 42.75% | 5,495 | 31.69% | 3,126 | | | 36.78% |
| **Comorbid diagnoses** |  |  |  |  |  |  |  | | |  |
| Psychotic disorder | 14,319 | 11.09% | 6,778 | 4.71% | 2,680 | 15.46% | 452 | | | 5.32% |
| SUD and Diabetes | 2,200 | 1.70% | 1,111 | 0.77% | 1,111 | 6.41% | 0 | | | 0.00% |
| Chronic disease | 25,331 | 19.62% | 31,671 | 21.99% | 5,845 | 33.72% | 5748 | | | 67.63% |
| **Sex** |  |  |  |  |  |  |  | | |  |
| Female | 71,755 | 55.58% | 81,094 | 56.31% | 8,301 | 47.88% | 4,703 | | | 55.34% |
| Male | 57,358 | 44.42% | 62,898 | 43.69% | 9,035 | 52.12% | 3,796 | | | 44.66% |
| **Age** |  |  |  |  |  |  |  | | |  |
| Mean age (SD) | 37.24 (12.12) | | 39 (11.89) | | 38.88(10.34) | | 48(9.88) | | | |
| **Race** |  |  |  |  |  |  |  | | |  |
| American Indian | 2,985 | 2.31% | 3,332 | 2.32% | 799 | 4.62% | 300 | | | 3.53% |
| Asian | 4,097 | 3.17% | 4,687 | 3.25% | 107 | 0.62% | 424 | | | 4.99% |
| Black | 29,331 | 22.72% | 29,082 | 20.19% | 2,525 | 14.54% | 1,581 | | | 18.60% |
| Multiracial | 3,257 | 2.52% | 2,880 | 1.97% | 368 | 2.09% | 126 | | | 1.48% |
| Pacific Islander | 171 | 0.13% | 211 | 0.15% | 15 | 0.09% | 23 | | | 0.27% |
| White | 76,769 | 59.46% | 90,397 | 62.70% | 12,340 | 71.13% | 4,880 | | | 57.42% |
| Race Missing | 12,503 | 9.68% | 13,403 | 9.43% | 1,182 | 6.91% | 1,165 | | | 13.71% |
| **Ethnicity** |  |  |  |  |  |  |  | | |  |
| Hispanic | 11,858 | 9.18% | 11,698 | 8.11% | 1,154 | 6.63% | 1,015 | | | 11.94% |
| Not Hispanic | 114,713 | 88.85% | 129,846 | 90.13% | 15,972 | 92.13% | 7,334 | | | 86.29% |
| Missing | 2,542 | 1.97% | 2,448 | 1.76% | 210 | 1.24% | 150 | | | 1.76% |
| **Education** |  |  |  |  |  |  |  | | |  |
| > High School | 73,711 | 57.09% | 80,258 | 55.69% | 10,255 | 59.12% | 4,452 | | | 52.38% |
| < High School | 21,801 | 16.89% | 26,464 | 18.39% | 3,440 | 19.85% | 1,558 | | | 18.33% |
| Missing | 33,601 | 26.02% | 37,270 | 25.92% | 3,641 | 21.03% | 2,489 | | | 29.29% |
| **Geography** |  |  |  |  |  |  |  | | |  |
| Urban | 90,479 | 70.08% | 92,310 | 64.10% | 11,223 | 64.66% | 5,288 | | | 62.22% |
| Rural | 29,858 | 23.13% | 31,327 | 21.77% | 3,573 | 20.59% | 1,885 | | | 22.18% |
| Missing | 8,776 | 6.80% | 20,355 | 14.13% | 2,540 | 14.74% | 1,326 | | | 15.60% |
| **Income** |  |  |  |  |  |  |  | | |  |
| ≤50% FPL | 82,077 | 63.57% | 104,860 | 72.82% | 14,560 | 83.99% | 5,659 | | | 66.58% |
| 50-100% FPL | 47,036 | 36.43% | 39,132 | 27.18% | 2,776 | 16.01% | 2,840 | | | 33.42% |
| >100% FPL | 0 | 0.00% | 0 | 0.00% | 0 | 0.00% | 0 | | | 0.00% |

Abbreviations: SUD, substance use disorder; SD, standard deviation; FPL, federal poverty level
