# Supplementary material for: Can telehealth expansion boost health care utilization specifically for patients with substance use disorders relative to patients with other types of chronic disease?
Source: PLoS One. 2024 Apr 1;19(4):e0299397. doi: 10.1371/journal.pone.0299397 (PMC10984462; doi:10.1371/journal.pone.0299397)
Supplement: S3 Appendix — (DOCX) [file pone.0299397.s003.docx]

**S3 Appendix. Definition of Outcome Measures**

We identified primary care visits for any diagnosis and primary care visits specifically for a SUD or diabetes diagnosis. We also specified whether the visit was in person or by telehealth.

Primary care visit

To identify primary care visits, as opposed to other kinds of outpatient visits, we used the following provider specialty codes from Wisconsin Badgercare documentation:
320 - geriatrics
316 – family practice
271 – general practice
318 – general practice
92 – nurse practitioner family practice

In addition, a number of provider specialty codes do not clearly indicate a specialty, or are a place of service without an associated specialty.
322 – internal medicine
100 – "Physician Assistant"
125 – "advanced practice nurse prescriber"
126 – "qualified treatment trainee"
184 - "hospital affiliated clinic"
185 - "free standing clinic"
300 - "free standing"
301 - "hospital affiliated"

To assess whether these codes represented primary care services, we cross-tabulated these codes with the rendering provider taxonomy. **Based on cross-tabulations, we included visits that were likely primary care** because either a) the visit occurs in a typical primary care setting, e.g. FQHC, CHC, Rural Health Center, etc., and neither the taxonomy nor the provider code indicate a non-primary care specialty, or b) the provider taxonomy indicated general practice (internal medicine or family medicine) along with a specialty often embedded in primary care practices (e.g., sleep medicine, sports medicine, obesity, addiction). In contrast, if the codes indicated internal medicine and a specialty such as pulmonology or cardiology then those visits were excluded. We also excluded visits for women’s health, family planning, and pediatrics

Finally, the following provider codes did not appear during our study period and were excluded: 80- FQHC, 81 -CHC, 83 – family planning, 93 – other NP, 95 – NP/nurse midwife.

Modality

We identified the use of telehealth, as opposed to in-person services, based on the presence of a procedure code, or the presence of either a place of service code or modifier indicating telehealth.

Modifier: GT or 95

Place of service indicator: POS 02

Procedure codes:

E-visits: 98970, 98971, 89872, 99421, 99422, 99443

Telephone E&M Services: 99441, 99442, 99443
