# Supplementary material for: Can telehealth expansion boost health care utilization specifically for patients with substance use disorders relative to patients with other types of chronic disease?
Source: PLoS One. 2024 Apr 1;19(4):e0299397. doi: 10.1371/journal.pone.0299397 (PMC10984462; doi:10.1371/journal.pone.0299397)
Supplement: S4 Appendix — (DOCX) [file pone.0299397.s004.docx]

**S4 Appendix. Regression models technical appendix**

| ***Independent Variables*** | ***1. Primary care utilization (Dependent Variable)*** | | |
| --- | --- | --- | --- |
|  | **Logit regression** | | |
|  | ***Coef*** | ***SE*** | ***p*** |
| Post-PHE x SUD | -0.068 | 0.018 | <0.001 |
| Post-PHE | -0.193 | 0.012 | <0.001 |
| SUD | 0.151 | 0.018 | <0.001 |
| Age | -0.003 | <0.001 | 0.151 |
| Female | 0.078 | 0.016 | <0.001 |
| ≤50 FPL | -0.163 | 0.017 | <0.001 |
| Hispanic | -0.038 | 0.038 | 0.323 |
| Ethnicity Missing | -0.181 | 0.065 | 0.005 |
| Rural | -0.040 | 0.021 | 0.063 |
| Geographical area missing | 0.322 | 0.018 | <0.001 |
| Psychotic Disorder Diagnosis | 0.103 | 0.025 | <0.001 |
| Chronic Condition | 0.156 | 0.017 | <0.001 |
| American Indian | 0.021 | 0.045 | 0.639 |
| Asian | 0.017 | 0.043 | 0.69 |
| Black | -0.122 | 0.022 | <0.001 |
| Multiracial | -0.040 | 0.060 | 0.507 |
| Pacific Islander | -0.066 | 0.169 | 0.697 |
| Race missing | 0.080 | 0.038 | 0.039 |
| Intercept | -2.551 | 0.043 | <0.001 |
| # Observations | 1,640,329 |  |  |
| Pseudo R-Sq | 0.0061 |  |  |

| ***Independent Variables*** | ***2. Telehealth utilization (Dependent Variable)*** | | |
| --- | --- | --- | --- |
|  | **Logit regression** | | |
|  | ***Coef*** | ***SE*** | ***p*** |
| Post-PHE x SUD | -1.506 | 0.343 | <0.001 |
| Post-PHE | 5.686 | 0.320 | <0.001 |
| SUD | 1.678 | 0.343 | <0.001 |
| Age | -0.006 | 0.002 | 0.007 |
| Female | 0.254 | 0.036 | <0.001 |
| ≤50 FPL | -0.219 | 0.038 | <0.001 |
| Hispanic | 0.066 | 0.078 | 0.394 |
| Ethnicity Missing | -0.409 | 0.162 | 0.012 |
| Rural | -0.272 | 0.053 | <0.001 |
| Geographical area missing | 0.312 | 0.042 | <0.001 |
| Psychotic Disorder Diagnosis | 0.226 | 0.057 | <0.001 |
| Chronic Condition | 0.158 | 0.039 | <0.001 |
| American Indian | 0.003 | 0.125 | 0.983 |
| Asian | 0.068 | 0.100 | 0.497 |
| Black | 0.019 | 0.052 | 0.715 |
| Multiracial | -0.351 | 0.114 | 0.002 |
| Pacific Islander | -0.098 | 0.454 | 0.829 |
| Race missing | 0.270 | 0.076 | <0.001 |
| Intercept | -9.910 | 0.334 | <0.001 |
| # Observations | 1,640,329 |  |  |
| Pseudo R-Sq | 0.1143 |  |  |

| ***Independent Variables*** | ***3. Fraction of telehealth utilization (Dependent Variable)*** | | |
| --- | --- | --- | --- |
|  | **Fracreg Logit regression** | | |
|  | ***Coef*** | ***SE*** | ***p*** |
| SUD | 0.109 | 0.045 | 0.014 |
| Age | -0.007 | 0.002 | 0.001 |
| Female | 0.198 | 0.041 | <0.001 |
| ≤50 FPL | -0.072 | 0.042 | 0.086 |
| Hispanic | 0.169 | 0.100 | 0.09 |
| Ethnicity Missing | -0.269 | 0.172 | 0.118 |
| Rural | -0.266 | 0.056 | <0.001 |
| Geographical area missing | -0.039 | 0.047 | 0.412 |
| Psychotic Disorder Diagnosis | 0.188 | 0.061 | 0.002 |
| Chronic Condition | 0.035 | 0.044 | 0.428 |
| American Indian | -0.035 | 0.134 | 0.796 |
| Asian | 0.052 | 0.106 | 0.623 |
| Black | 0.149 | 0.059 | 0.012 |
| Multiracial | -0.379 | 0.129 | 0.003 |
| Pacific Islander | 0.051 | 0.450 | 0.91 |
| Race missing | 0.262 | 0.099 | 0.008 |
| Intercept | -1.070 | 0.117 | <0.001 |
| # Observations | 50,411 |  |  |
| Pseudo R-Sq | 0.009 |  |  |
