# Supplementary material for: Can telehealth expansion boost health care utilization specifically for patients with substance use disorders relative to patients with other types of chronic disease?
Source: PLoS One. 2024 Apr 1;19(4):e0299397. doi: 10.1371/journal.pone.0299397 (PMC10984462; doi:10.1371/journal.pone.0299397)
Supplement: Attachment — Submitted filename: Response to reviewers_122623.docx [file pone.0299397.s006.docx]

Dear Dr. Ray and the Editorial Board of PLOS ONE,

Thank you for your recent feedback on our submitted manuscript, “Can telehealth expansion boost health care utilization specifically for patients with substance use disorders relative to patients with other types of chronic disease?” We are pleased to submit a revised version. As requested, we have provided a point-by-point response to both reviewers’ comments and completed the necessary formatting and file adjustments. Thank you in advance for considering the revised version of our manuscript. We feel the article is improved through this revision process.

Sincerely,

Alyssa Shell Tilhou, MD, PhD

Boston University Medical Center

Response to Reviewers

1. If the authors have adequately addressed your comments raised in a previous round of review and you feel that this manuscript is now acceptable for publication, you may indicate that here to bypass the “Comments to the Author” section, enter your conflict of interest statement in the “Confidential to Editor” section, and submit your "Accept" recommendation.

Reviewer #1: All comments have been addressed

Reviewer #2: All comments have been addressed

2. Is the manuscript technically sound, and do the data support the conclusions?

The manuscript must describe a technically sound piece of scientific research with data that supports the conclusions. Experiments must have been conducted rigorously, with appropriate controls, replication, and sample sizes. The conclusions must be drawn appropriately based on the data presented.

Reviewer #1: Yes

Reviewer #2: Yes

3. Has the statistical analysis been performed appropriately and rigorously?

Reviewer #1: Yes

Reviewer #2: Yes

4. Have the authors made all data underlying the findings in their manuscript fully available?

The [PLOS Data policy](http://www.plosone.org/static/policies.action#sharing) requires authors to make all data underlying the findings described in their manuscript fully available without restriction, with rare exception (please refer to the Data Availability Statement in the manuscript PDF file). The data should be provided as part of the manuscript or its supporting information, or deposited to a public repository. For example, in addition to summary statistics, the data points behind means, medians and variance measures should be available. If there are restrictions on publicly sharing data—e.g. participant privacy or use of data from a third party—those must be specified.

Reviewer #1: No

*We have included the requested data availability statement. Please let us know if further commentary is needed.*

Reviewer #2: Yes

5. Is the manuscript presented in an intelligible fashion and written in standard English?

PLOS ONE does not copyedit accepted manuscripts, so the language in submitted articles must be clear, correct, and unambiguous. Any typographical or grammatical errors should be corrected at revision, so please note any specific errors here.

Reviewer #1: Yes

Reviewer #2: Yes

6. Review Comments to the Author

Please use the space provided to explain your answers to the questions above. You may also include additional comments for the author, including concerns about dual publication, research ethics, or publication ethics. (Please upload your review as an attachment if it exceeds 20,000 characters)

Reviewer #1: (No Response)

Reviewer #2: The revision addresses the points I raised on the initial submission. I have a few formatting/presentation suggestions:

Discussion of Fig 1: I cannot see on the figure that diabetes initially had more visits (may be that the chosen colors make it hard to differentiate?) (around line 212)

We have corrected the wording in this section to clarify our intent and compare visits across individuals in the diabetes and SUD cohort (line 213 of the revised manuscript).

*“Of cohort members completing visits in the week post-PHE, a slightly greater percentage of the diabetes cohort initially completed visits via telehealth relative to the SUD cohort.”*

Table 1: Didn’t you add other comorbid conditions that would make more sense to show in the Comorbid diagnosis panel other than “SUD and diabetes”?

Chronic disease comorbidities has been added as a row to Table 1. Thank you for this correction.

Table 1: consider removing “missing sex” row? (because it is 0 for all?)

*Completed (including in the appendix).*

Table 1: consider removing “missing income” row? (because it is not missing for any?)

*Completed (including in the appendix).*

Figure 1: change label to read “in person” not IP? (Some health econ readers might infer IP=inpatient?)

*Completed.*

Titles to Fig 1 and 2: I think you need to add “diagnosis”

*Completed.*

Parentheses to close on line 200

*Completed.*

Exhibiting not exhibited on line 202

*Completed.*

7. PLOS authors have the option to publish the peer review history of their article ([what does this mean?](https://journals.plos.org/plosone/s/editorial-and-peer-review-process#loc-peer-review-history)). If published, this will include your full peer review and any attached files.

If you choose “no”, your identity will remain anonymous but your review may still be made public.

**Do you want your identity to be public for this peer review?** For information about this choice, including consent withdrawal, please see our [Privacy Policy](https://www.plos.org/privacy-policy).

Reviewer #1: No

Reviewer #2: No

[NOTE: If reviewer comments were submitted as an attachment file, they will be attached to this email and accessible via the submission site. Please log into your account, locate the manuscript record, and check for the action link "View Attachments". If this link does not appear, there are no attachment files.]

While revising your submission, please upload your figure files to the Preflight Analysis and Conversion Engine (PACE) digital diagnostic tool, <https://pacev2.apexcovantage.com/>. PACE helps ensure that figures meet PLOS requirements. To use PACE, you must first register as a user. Registration is free. Then, login and navigate to the UPLOAD tab, where you will find detailed instructions on how to use the tool. If you encounter any issues or have any questions when using PACE, please email PLOS at [figures@plos.org](mailto:figures@plos.org). Please note that Supporting Information files do not need this step.

*Configuration confirmed on PACE.*

*In compliance with data protection regulations, you may request that we remove your personal registration details at any time.* [*(Remove my information/details)*](https://www.editorialmanager.com/pone/login.asp?a=r)*. Please contact the publication office if you have any questions.*
